# Antigen specificity and inflammatory amplification in autoimmune hepatitis

**DOI:** 10.3389/fimmu.2026.1996626

**Published:** 2026-10-19

**Authors:** Zihao Xu, Yifan Liu, Haoyang Zou, Sheng Guo

**Affiliations:** 1 School of Chinese Medicine, Hubei University of Chinese Medicine, Wuhan, Hubei, China; 2 Institute of Liver Diseases, Hubei Key Laboratory of Theoretical and Applied Research of Liver and Kidney in Traditional Chinese Medicine, Hubei Provincial Hospital of Traditional Chinese Medicine, Wuhan, China; 3 Hubei Provincial Clinical Research Center of Traditional Chinese Medicine for Liver Diseases, Hubei, Wuhan, China; 4 Hubei Shizhen Laboratory, Hubei, Wuhan, China; 5 Affiliated Hospital of Hubei University of Chinese Medicine, Hubei, Wuhan, China

**Keywords:** antigen presentation, autoantigens, autoimmune Hepatitis, HLA, liver inflammation, SEPSECS, T-cell receptor

## Abstract

Human leukocyte antigen (HLA) associations anchor genetic susceptibility to autoimmune hepatitis (AIH). Connecting this susceptibility to the immune reactions that sustain liver injury remains a central challenge. Recent antigen-specific experiments and tissue analyses provide complementary insights into recognition and injury. This Mini Review examines how population-specific HLA associations, SepSecS-reactive lymphocytes and intrahepatic immune states inform the relationship between antigen recognition and inflammatory amplification. The evidence reveals three unresolved relationships between susceptibility, recognition and injury. Class II-restricted autoreactivity and conditionally independent class I associations in Han Chinese AIH implicate potentially complementary immune pathways. Antigen-specific T-cell responses extend beyond the boundaries defined by autoantibody status. Receptor specificity and the capacity to sustain tissue injury require distinct functional tests. We evaluate antigen-presentation experiments, receptor studies and tissue profiling, distinguishing human observations from mechanistic models. The synthesis supports a model in which heterogeneous antigen-specific responses may converge on shared inflammatory processes. Priorities include linking genetic risk to natural antigen presentation and defining autoreactive clone function in liver tissue. Matched perturbation experiments should then test antigen dependence alongside cytokine-dependent injury. Resolving these questions would clarify which mechanisms require antigen-specific intervention and which reflect downstream inflammatory amplification.

## 1 Introduction

Autoimmune hepatitis (AIH) combines strong major histocompatibility complex (MHC) associations with experimentally identifiable autoreactive lymphocytes (1–5). Current models connect antigen presentation with B- and T-cell effector activity and deficient immune regulation (6, 7). Genetic studies define susceptibility at population scale, while antigen-focused experiments resolve recognition by individual clones. Tissue studies address immune activity at the site of injury. These approaches investigate related biological processes, but their conclusions concern different stages of disease.

The central question is how antigen recognition contributes to sustained hepatic inflammation. A risk-associated human leukocyte antigen (HLA) allele defines susceptibility, an autoreactive clone establishes recognition, and a functional tissue assay tests injury. Connecting these levels requires evidence from complementary experimental systems. Treating these observations as successive steps in one obligatory pathway can obscure alternative mechanisms and overstate the completeness of the evidence.

This focused narrative Mini Review examines HLA susceptibility, antigen-specific immunity and inflammatory amplification in AIH. We emphasize AIH-1 and retain AIH-2 as a distinct antigen-defined comparator. The synthesis draws on published genetic, antigen-specific and tissue studies, supplemented by targeted citation searches updated on 30 September 2026. It compares mechanistic findings and competing interpretations across these experimental settings. Three questions guide the discussion: how HLA risk relates to recognition, what autoreactive clones reveal about tissue disease, and which experiments can distinguish antigen dependence from inflammatory amplification. **Figure 1** organizes these findings into genetic susceptibility, antigen recognition and inflammatory amplification, highlighting the experimental connections within each domain.

**Figure 1:**
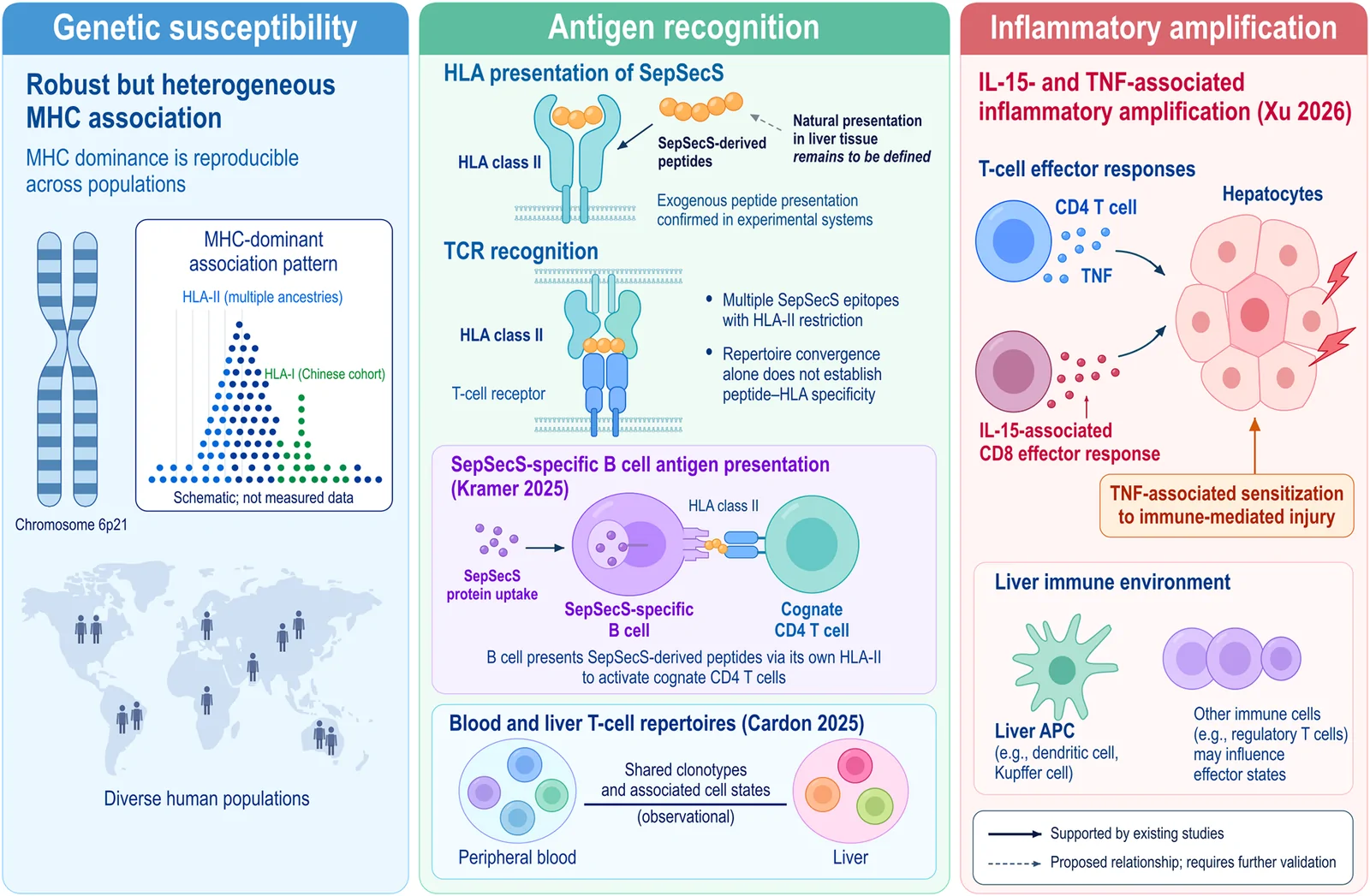
Genetic susceptibility, antigen recognition and inflammatory amplification in autoimmune hepatitis. Left, MHC associations vary across populations, with prominent HLA-II associations and conditionally independent HLA-I signals identified in Han Chinese studies (1–3, 8). The association plot and population map are conceptual illustrations, not measured data or a map of study recruitment. Center, SepSecS studies establish peptide–HLA-II recognition and antigen-specific B-cell presentation to cognate CD4 T cells (4, 5, 11). Presentation of supplied peptides and processing of soluble protein in experimental systems are distinguished from the identification of naturally presented ligands in liver tissue. Human antigen-focused profiling links circulating autoreactive clonotypes with liver-associated repertoires and cell states (29). The undirected blood–liver connector denotes observational relationships, without assigning a direction of migration. Right, functional and tissue studies implicate IL-15-associated CD8 effector responses and CD4-derived TNF in inflammatory amplification and hepatocyte susceptibility (30). The red particles represent effector activity rather than CD8-derived IL-15. The panel summarizes an injury network and does not assign SepSecS specificity to its effector cells. Antigen-presenting cells and regulatory populations provide additional tissue context. Solid arrows denote relationships supported in the cited experimental settings; dashed arrows indicate unresolved links. The three domains organize complementary evidence rather than a single validated causal sequence in the same patients. APC, antigen-presenting cell; HLA, human leukocyte antigen; IL-15, interleukin-15; MHC, major histocompatibility complex; TCR, T-cell receptor; TNF, tumor necrosis factor.

## 2 HLA susceptibility does not specify a single antigenic pathway

MHC dominance is more reproducible than the identity of the leading HLA association. European AIH-1 data principally implicate HLA-DRB1*03:01 and HLA-DRB1*04:01 (1). Chinese genome-wide and fine-mapping studies identify a different architecture, including conditionally independent HLA-B*35:01, HLA-B*08:01 and rs7765379 signals (2, 3). Japanese studies further distinguish DRB1 and DQB1 configurations associated with disease (8). Across populations, the shared finding is a major MHC contribution with population-specific susceptibility profiles.

This heterogeneity leaves two interpretations open. Different populations may carry distinct antigen-presentation pathways, or different allele frequencies and linkage structures may expose different markers of related biology. HLA imputation and conditional analysis are also sensitive to reference-panel composition and local dependence (9, 10). A class I association consequently motivates investigation of class I biology without proving a CD8-mediated mechanism. Comparisons must first reconcile ancestry, phenotype and typing resolution before assigning population differences to distinct immune pathways.

Statistical refinement should also be distinguished from independent replication. The Han Chinese fine-mapping analysis examined 1,622 cases and 10,466 controls from the genome-wide study context (2, 3). It refines molecular resolution within the existing population-level evidence. Shared participants should therefore be accounted for when weighing genetic evidence against smaller functional studies.

The gap between class I genetics and class II antigen experiments is particularly informative. SepSecS-specific CD4 responses provide direct evidence of HLA-II-restricted recognition (4, 5, 11). Those experiments do not functionally explain the conditionally independent HLA-B associations in Han Chinese AIH (3). Cooperation between class I-mediated recognition and class II-mediated help is one testable explanation for these complementary findings. The useful question is whether a risk-associated molecular feature changes a relevant immune response under controlled HLA conditions.

HLA risk also exists within a broader susceptibility architecture. The Chinese genome-wide meta-analysis identified non-MHC loci near CD28–CTLA4–ICOS and SYNPR, with functional experiments supporting regulation of CTLA4 (2). These findings provide a reason to consider immune regulation alongside peptide binding. Serological subgroup associations, including those reported in anti-SLA/LP-positive Chinese patients, further complicate comparisons (12). Antigen-presentation models should therefore incorporate regulatory susceptibility and serological subgroup composition.

Clinical heterogeneity further limits the interpretation of a common disease label. Adult and pediatric genetic evidence covers different populations and disease presentations (13). Age at onset and autoantibody profile should therefore accompany ancestry in comparisons of risk architecture. A subgroup association may nominate an informative stratum without establishing a mechanistically distinct disease. The distinction matters when recruiting antigen-selected cohorts to explain population-wide susceptibility.

## 3 Antigen-specific experiments reveal both convergence and heterogeneity

### 3.1 SepSecS connects B-cell recognition with CD4 responses

Soluble liver antigen/liver-pancreas (SLA/LP) antibodies recognize SepSecS, a molecularly defined autoantigen identified through recombinant antigen studies (14, 15). Standardized assays established strong disease specificity, although antibody positivity characterizes only a subset of AIH (16). Epitope mapping subsequently identified HLA-DRB1*03:01-restricted CD4 responses using transgenic mice and selected patient samples (11). These studies transformed a serological marker into an experimentally accessible recognition system. Their mechanistic scope centers on SepSecS-associated immunity in selected disease settings.

Clonal analysis clarifies a possible role for antigen-specific B cells beyond antibody secretion. Kramer and colleagues isolated affinity-matured SepSecS-reactive antibodies and multiple SepSecS-specific CD4 clones (4). SepSecS-specific B cells presented soluble antigen to cognate T cells more effectively than B cells of unrelated specificity. This directly supports antigen-selective B-cell presentation in the tested system. This interaction offers a plausible mechanism for maintaining coordinated B- and T-cell responses.

Seronegativity is an incomplete boundary for this cellular response. The same study detected SepSecS-reactive CD4 clones in anti-SLA-negative AIH and, at lower levels, in non-AIH liver disease and healthy individuals (4). These findings favor measuring cellular recognition directly and comparing its magnitude and phenotype with appropriate controls. Frequency, differentiation and tissue behavior may distinguish disease-associated responses more effectively than a binary antigen-reactivity label.

Comparison with earlier work reveals a substantive detection problem. Renand and colleagues detected SLA-reactive CD4 cells only in anti-SLA-positive patients and identified a pro-inflammatory, B-helper profile (17). Their combined single-cell and receptor analyses localized autoreactive clonotypes within a memory PD-1-positive, CXCR5-negative compartment. The broader detection in the later clonal study need not imply a biological contradiction (4). Different assays and sampling strategies may recover distinct parts of a rare response. Direct comparison in the same samples would distinguish a serologically restricted population from incomplete detection.

Earlier Chinese epitope work also examined cellular recognition across antibody groups. Zhao and colleagues measured interferon-γ responses to overlapping SLA peptides in 31 AIH-1 patients (18). Antibody-positive patients had more frequent and stronger responses than antibody-negative patients. These results support an association between humoral and cellular reactivity without making them equivalent. Together, the studies argue for retaining quantitative response measurements when defining antigen-associated patient groups.

Recent receptor reconstruction broadens the HLA context of SepSecS recognition. Guinebretière and colleagues tested 17 paired T-cell receptors (TCRs) derived from five patients and identified six reactive regions with four class II restrictions (5). The results included non-HLA-DR presentation and a shared CDR3 motif associated with an HLA-DP-restricted epitope. These assays identify several peptide–HLA–TCR combinations under exogenous peptide-loading conditions. They do not establish that the corresponding ligands are naturally generated from hepatic SepSecS and presented at sufficient density in patients, or that their recognition causes liver injury.

An HLA molecule that presents an epitope need not be a disease-risk allele. Restriction describes the molecular conditions of recognition, whereas association compares susceptibility between groups. The SepSecS experiments establish the former much more directly than the latter (5, 11). Likewise, a common motif among selected antigen-reactive receptors does not estimate its prevalence in an unselected AIH population. Connecting epitope maps to genetic susceptibility therefore requires comparison with population-level risk estimates.

### 3.2 Other antigen systems constrain a universal model

CYP2D6 provides a separate example of defined recognition in AIH-2. Patient T-cell responses span multiple epitopes and associate with HLA-DRB1*07 and disease activity (19). The antibody and genetic context of AIH-2 differs from that of AIH-1 (20, 21). Therefore, CYP2D6 supports the feasibility of resolving antigen-specific responses without supplying a transferable explanation for all AIH. Its interpretation depends on the subtype, HLA background and experimental setting.

New humoral findings extend the antigenic landscape. Klepper and colleagues identified AIH-associated responses to DIP2A and the relaxin receptor RXFP1 using phage-display profiling (22). Competition experiments supported antibody cross-reactivity between DIP2A and a human herpesvirus 6 peptide. Patient IgG also inhibited relaxin-2 signaling in an RXFP1 assay. These observations distinguish candidate microbial cross-reactivity from receptor-directed functional effects; neither establishes viral initiation or HLA-restricted T-cell causality in patients.

Together, these systems reveal distinct functional roles for AIH-associated autoantigens. A target can be a diagnostic marker, a substrate for B-cell antigen presentation, or a participant in a functional antibody response. Recognition by a T cell introduces another distinct evidentiary requirement. The mechanistic status of each antigen therefore depends on the assay and biological outcome demonstrated. Antigen discovery gains mechanistic meaning when binding, presentation and effector function are evaluated separately.

### 3.3 Natural processing remains distinct from peptide recognition

Antigen-processing and peptide-recognition experiments resolve complementary stages of the immune response. Presentation of soluble SepSecS by antigen-specific B cells supports uptake and processing in a defined cellular system (4). Peptide-based TCR reconstruction resolves recognition and HLA restriction under controlled conditions (5). Exogenous peptide supply bypasses physiological antigen uptake and proteolytic processing and can permit recognition at ligand densities not established *in vivo*. A reactive receptor therefore demonstrates recognition capacity, rather than proving encounter with a naturally processed, liver-derived autoantigen in human disease. Establishing that connection requires recognition of cells processing intact antigen under appropriate HLA conditions, followed by evidence of the cognate ligand and relevant clone function in patient liver tissue.

Immunopeptidomics addresses this availability question by detecting HLA-associated peptides (23). Its interpretation depends on cell source, recovery and identification confidence. Detection would support natural presentation but would not establish that the responding clone causes damage. Failure to detect a peptide would also be inconclusive without an adequate sensitivity assessment. The decisive next step is to connect naturally available ligands with disease-relevant cellular functions.

## 4 Receptor identity and liver injury require different evidence

### 4.1 Clonal sharing becomes informative when specificity is assigned

AIH repertoires contain both private clonotypes and recurring features. Early liver studies described clonal expansion without a complete CDR3 sequence shared by every patient (24, 25). Higher-throughput sequencing subsequently identified repertoire biases, persistent abnormalities during remission and shared liver TCR clusters (26). These findings are compatible because complete sequence identity, motif similarity and V/J usage measure different properties. Shared receptor features prioritize candidates for functional antigen-specificity testing in defined HLA contexts.

Single-cell pairing can connect receptor sequence with transcriptional state, but state annotation cannot independently assign specificity (27, 28). Cardon and colleagues advanced this connection through antigen-focused single-cell profiling and comparison with expanded intrahepatic clonotypes in autoimmune liver disease (29). Their human data supported clonal overlap and similarities in cell state between circulating autoreactive CD4 cells and intrahepatic populations. These observations do not establish whether cells entered or exited the liver. Mechanistic experiments used a mouse model with a defined hepatic antigen. The human observations link compartments without resolving the initiating antigen, the direction of trafficking or the relationship to population HLA risk.

Each matching approach resolves a distinct aspect of the tissue response. A shared blood–liver clonotype establishes a clonal relationship but does not determine migration direction. A tissue-associated transcriptional signature indicates a cellular program but does not establish its antigenic cause. Reconstructed receptor recognition tests specificity under the supplied assay conditions. Combining these observations is most informative when the same clone is followed across assays, with explicit separation of patient observations from experimental inference.

Persistence during remission is especially easy to overinterpret. The repertoire abnormalities observed after treatment demonstrate that immunological normalization and biochemical remission are not equivalent (26). They do not establish that every persistent clone remains pathogenic. A retained clone could have altered function, reduced antigen exposure or a memory phenotype. Longitudinal analyses should consequently compare clone-specific activity with disease state, instead of treating persistence itself as a surrogate for ongoing injury.

### 4.2 Inflammatory context changes the consequences of recognition

Tissue studies now provide direct mechanisms of inflammatory amplification. Xu and colleagues combined AIH multi-omics with functional experiments and an open-label phase IIa trial (30). Their results implicated IL-15 in promoting auto-aggressive CD8 responses and CD4-derived tumor necrosis factor (TNF) in supporting cytotoxicity and hepatocyte susceptibility. These findings advance the downstream injury mechanism without identifying the antigen recognized by each expanded clone. The trial provides a clinical rationale for further investigation of TNF-dependent disease activity.

Immune regulation admits competing interpretations. Taubert and colleagues found intrahepatic regulatory T-cell enrichment in untreated AIH and higher regulatory-to-effector and regulatory-to-B-cell ratios in patients achieving remission (31), consistent with a compensatory protective response. Studies of impaired CD39-positive suppression, costimulatory imbalance and regulatory dysfunction instead support inadequate control of inflammation (32–34). Kwon and colleagues observed increased intrahepatic regulatory populations alongside reduced suppression in assays using peripheral blood cells (34); the functional assay does not directly establish that the expanded intrahepatic cells cause injury. Expansion may reflect compensation for inflammation, while impaired suppression may permit its persistence. Neither abundance nor correlation with disease activity distinguishes these possibilities. Tissue-matched functional measurements are needed to separate protective recruitment, insufficient regulation and acquisition of inflammatory function, accounting for treatment and disease stage.

Functional perturbation provides stronger evidence than a count alone. In patient-derived cultures, Longhi and colleagues suppressed the IL-17-associated program during generation of regulatory cells from CD25-negative precursors (35). The resulting cells showed improved suppressive activity and phenotypic stability. These experiments establish modifiable regulatory function in cells generated under defined culture conditions. This supports testing the state and stability of regulation alongside the specificity of effector recognition.

Hepatic stellate-cell responses add a fibrogenic dimension to the consequences of persistent liver injury. In a study of chronic hepatitis B-associated fibrosis, PR/SET domain 1 (PRDM1) overexpression promoted stellate-cell ferroptosis and reduced activation through regulation of glutathione peroxidase 4 (GPX4) (36). In transforming growth factor-β1-stimulated LX-2 cells, embryonic lethal abnormal vision like 1 (ELAVL1)-mediated stabilization of histone deacetylase 6 (HDAC6) was linked to reduced ribosomal protein S5 (RPS5) acetylation and enhanced stellate-cell activation (37). These studies illustrate downstream remodeling mechanisms in other liver-disease settings; their contribution to AIH requires disease-specific validation.

These findings favor separating recognition from its inflammatory consequences. Antigen-specific activation could contribute to a tissue environment that permits additional effector activity. Alternatively, several antigen-specific pathways could converge on similar downstream states. Both interpretations accommodate diversity in genetic and receptor observations, but neither establishes that antigen recognition becomes dispensable during chronic disease. **Table 1** summarizes the observations that distinguish these levels and the causal questions they leave open.

**Table 1:** Evidence separating susceptibility recognition and injury.

Evidence	Established observation	Interpretation boundary	Next discriminating test
European and Chinese genetics (1–3)	Strong MHC associations with differing leading signals	Conditional independence is statistical, not proof of independent mechanisms	Compare candidate molecular effects under controlled HLA conditions
SepSecS clonal experiments (4)	Specific B cells present soluble antigen to reactive CD4 clones	Antigen presentation does not demonstrate antibody-mediated killing	Test the contribution of B-cell presentation to tissue effector responses
SepSecS TCR reconstruction (5)	Several epitopes and HLA-II restrictions support recognition	Peptide-loading assays do not establish natural liver presentation	Verify processing and presentation of the recognized ligand
Antigen-focused single-cell analysis (29)	Human clonal and state links; separate mouse mechanistic evidence	Clonal overlap does not determine migration direction or establish the initiating antigen	Follow functionally assigned clones across compartments
AIH injury network (30)	Functional support for IL-15 and TNF-dependent amplification	Downstream activity does not explain the initiating HLA–antigen interaction	Compare antigen-presentation and cytokine perturbations
DIP2A and RXFP1 antibodies (22)	Cross-reactive binding and inhibition of receptor signaling in assays	Neither result proves human disease initiation	Establish temporal order and disease-relevant function

HLA, human leukocyte antigen; MHC, major histocompatibility complex; TCR, T-cell receptor; TNF, tumor necrosis factor. Proposed tests are research priorities, not reported findings.

The competing interpretations make different predictions. Continued dependence on a dominant antigen would predict reproducible coupling between its validated cellular response and injury across disease states. Convergence of several antigenic pathways would instead predict more consistent downstream activity than shared receptor identity. A primarily regulatory explanation would predict functional differences despite comparable recognition. These predictions guide experimental comparisons, allowing for cooperation among antigenic and regulatory processes.

## 5 Discussion

The evidence supports a more differentiated question than whether AIH has an antigen-specific basis. Defined recognition is demonstrable, yet its relationship to genetic susceptibility and sustained injury remains unevenly resolved. Our interpretation is that antigen selection, immune regulation and inflammatory amplification should be analyzed as interacting processes. The available findings accommodate diverse antigenic inputs together with a continuing requirement for antigen-specific stimulation.

The Han Chinese HLA-B associations require particular care in this interpretation. The fine-mapping study used a Han-MHC reference panel of 10,689 individuals, reducing broad ancestry mismatch (3). Nevertheless, reference-panel size, allele representation and haplotype coverage influence imputation accuracy, especially for less frequent alleles (9, 10). Differences in these features can complicate comparisons across populations. Extensive linkage disequilibrium also allows an associated HLA-B allele to tag correlated coding or regulatory variation. Conditional independence is therefore relative to the variants measured and included in the model; it does not establish separate causal immune mechanisms. Direct high-resolution typing and replication in independent, ancestry-matched cohorts would help assess residual imputation and linkage effects before assigning the signals to distinct antigen-presentation pathways.

Three comparisons would most directly clarify this relationship. First, risk HLA carriers and suitable noncarriers should be compared for natural presentation of candidate antigens. HLA typing, peptide detection and receptor assays would address complementary parts of this test (9, 10, 23). Positive peptide binding alone would be insufficient. Conversely, nondetection would weaken a candidate only when recovery, HLA expression and assay sensitivity were adequate. This comparison would test how risk-associated HLA molecules shape presentation of SepSecS and other candidate antigens.

Second, validated antigen-reactive clones should be examined across blood and clinically obtained liver samples. Receptor reconstruction and state profiling would determine whether the same specificity accompanies distinct functional states (4, 5, 27). Untreated, treated and relapsing samples could help separate persistent recognition from changes in inflammatory activity. Donor identity must remain the unit of biological replication; group-pooled cells cannot estimate between-patient variability (38). Interpretation should account for biopsy indication, treatment exposure and donor-level variation.

Third, matched functional systems should compare interruption of cognate presentation with interruption of inflammatory signaling. A response dependent on both would support cooperation between recognition and amplification. Persistence of injury after disrupting one tested antigenic interaction would instead motivate examination of alternative antigens or downstream effectors. Such a result would not, by itself, prove global antigen independence. Comparing these perturbations would help resolve causal dependencies suggested by observational molecular networks.

These priorities also define appropriate roles for integrative bioinformatics. HLA fine-mapping can nominate molecular candidates, and receptor analysis can organize experimentally testable specificity groups (10, 28). Integration should preserve donor, antigen, HLA restriction and tissue identity to support mechanistic comparisons. Small antigen-selected cohorts and uneven ancestry coverage currently restrict generalization. The present narrative synthesis identifies experimentally supported connections and specifies the comparisons needed to resolve interpretations across studies.

The immediate opportunity is to connect established recognition systems with experimentally defined tissue functions. Success would mean showing when an antigen-specific response is required for injury and when shared inflammatory processes account for its persistence. That distinction could sharpen the rationale for antigen-directed versus pathway-directed interventions. It would also convert the robust HLA association from a general susceptibility marker into a more specific account of immune dysfunction.
